# Uncommon foreign body in the hypopharynx: A case report

**DOI:** 10.1097/MD.0000000000011242

**Published:** 2018-06-29

**Authors:** Guowei Chen, Yizhen Luo, Hongguang Pan, Yishu Teng, Zhenjiang Liang, Lan Li

**Affiliations:** a Department of Otolaryngology; b Department of Radiology, Shenzhen Children's Hospital, Shenzhen, Guangdong, China.

**Keywords:** flexible laryngoscopy, foreign body, hypopharynx, infant, size tag

## Abstract

**Rationale:**

Pharyngeal foreign bodies are a common complaint in emergent cases, and sometimes can be lethal. A large variety of foreign bodies may lodge in the pharynx. We present a case of uncommon foreign body in the hypopharynx.

**Patient concerns:**

A 9-month-old boy presented for an hour of crying associated with having congee. No abnormality was revealed in physical examination or cervicothoracic computed tomography (CT) scan. Flexible laryngoscopy showed a size tag on the posterior hypopharyngeal wall.

**Diagnoses:**

Foreign body in the hypopharynx.

**Interventions:**

The foreign body was removed from the hypopharynx.

**Outcomes:**

The patient was discharged after the removal of the foreign body.

**Lessons:**

Infants with foreign bodies in the hypopharynx may presented with no obvious clinical manifestation. Pharyngeal foreign bodies may be the potential for disastrous consequences. Otolaryngologists should pay attention to these foreign bodies.

## 1 Introduction

Pharyngeal foreign bodies are a common complaint frequently faced by otolaryngologists in medical emergencies. Ingestion of foreign body can occur among all age groups. Older children and adults seeking medical consultations may complain of odynophagia and throat pain. However, infants and younger children may present after vomiting, drooling, or coughing noticed by their caregivers. All kinds of foreign bodies may lodge in the pharynx. In China, fish bones and other animal bones are the most common pharyngeal foreign bodies.^[1]^ Some uncommon foreign bodies may lodges in the pharynx, such as pencil and barbecue blunder.^[2,3]^ Size tags are common items in daily human life. However, there have been no cases of ingestion of these foreign bodies reported. Here, we report an uncommon case of a size tag which was swallowed by a boy with no obvious presenting symptoms and lodged on the posterior hypopharyngeal wall.

The publication of a case report is exempted from approval by the Institutional Review Board of Shenzhen Children's Hospital. However, informed consent was obtained from this patient's parents for the publication of this case report.

## 2 Case report

**Figure 1:**
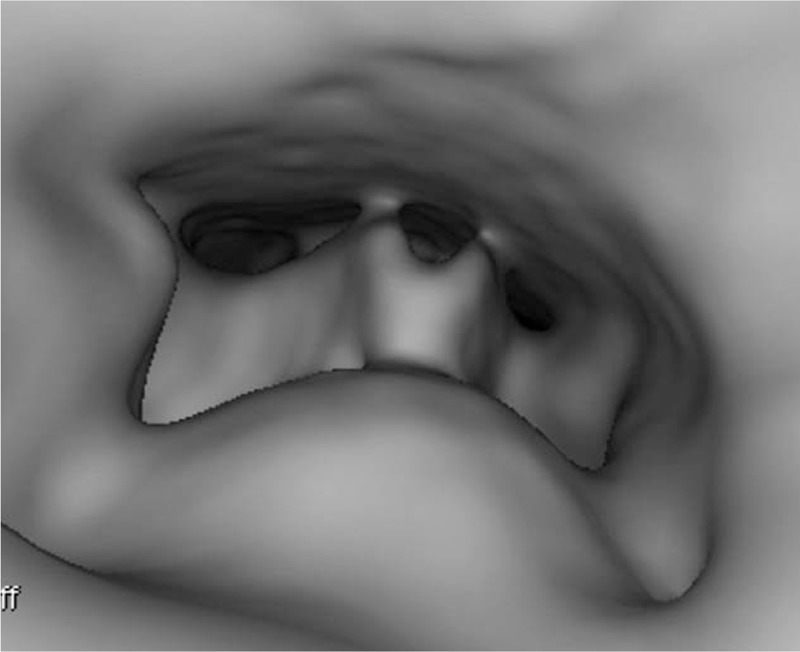
Virtual endoscopy of the hypopharynx.

**Figure 2:**
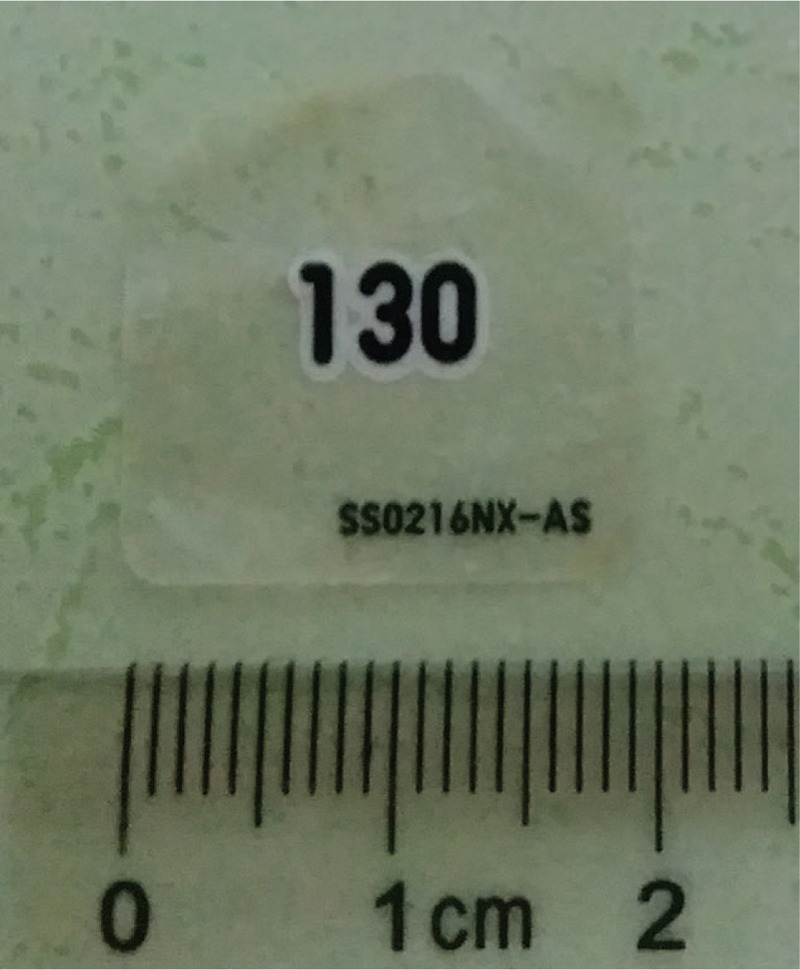
Foreign body removed from the hypopharynx.

**Figure 3:**
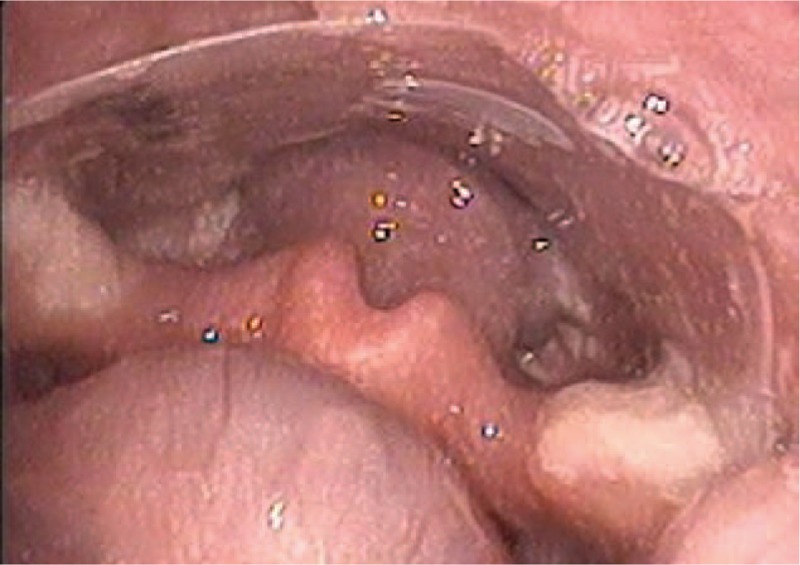
Foreign body in the hypopharynx.

A 9-month-old boy presented to Department of Otolaryngology for an hour of crying associated with having congee. He was suspected of foreign body aspiration. However, there were no suggestive symptoms, such as dyspnea, stridor, croupy cough, hoarseness, or vomiting. In physical examination, no foreign body was found in the oropharynx. Differences in the intensity, phase, and quality of bilateral breath sounds were uncertain for disturbance of baby crying. Therefore, cervicothoracic computed tomography (CT) scan with virtual endoscopy was taken for excluding foreign bodies of the pharynx, larynx, trachea, bronchi, and esophagus. However, no abnormality was revealed in the CT scan, as shown in Fig. 1. Then, flexible laryngoscopy was applied for viewing the hypopharynx and larynx. A transparency film, exhibited in Fig. 2, was visualized on the posterior hypopharyngeal wall, as shown in Fig. 3. The removal of the foreign body was proceeded immediately. The tongue was depressed for better exposure of the foreign body. Under direct visualization it was removed with forceps.

## 3 Discussion

A large variety of foreign bodies may lodge in the pharynx. In China, the majority of pharyngeal foreign bodies are food ingredients. By far fish bones and other animal bones are the most common objects. The locations for foreign bodies lodged are mainly in the palatine tonsils, base of the tongue, valleculae, and pyriform sinuses.^[1,4,5]^ Size tags are common items in daily human life. They are always attached to new garments. To our knowledge, there has been no case of a size tag in the hypopharynx reported.

For medical consultations, older children and adults may complain of odynophagia and throat pain. Infants can not communicate, they may present after vomiting, drooling, or coughing witnessed by their caregivers. However, the boy in the present case was too young to communicate. He visited Department of Otolaryngology for crying after having congee. Parents complained that the boy might had aspirated a foreign body, but he presented no obvious clinical manifestation. It was difficult to make the exact diagnosis at that moment. Auxiliary examinations were applied immediately.

In the present case, the size tag is a translucency film. It stuck on the posterior hypopharyngeal wall. The baby had no symptoms except crying. Cervicothoracic CT scanning revealed no positive results. However, negative results of imaging did not definitely rule out the presence of the foreign body.^[6]^ Flexible laryngoscopy was proceeded in the pharyngeal and laryngeal examination. It provided a better view of the hypopharynx and larynx, and helped to discover a size tag which lodged on the posterior hypopharyngeal wall. The size tag was a thin film and attached to the posterior hypopharyngeal wall. It may be the reason we could not discover the foreign body with cervicothoracic CT scanning.

Most foreign bodies in the oropharynx can be seen and be removed easily. However, foreign bodies in the hypopharynx are not easily discovered. They may be the potential for disastrous consequences. When they are big enough, they may block the esophagus, larynx, or lower respiratory tract and cause vomiting, suffocation, or death. Otolaryngologists should pay attention to these foreign bodies.

In conclusion, we present a case of uncommon foreign body in the hypopharynx with no obvious clinical manifestation.

## Author contributions

**Conceptualization:** Guowei Chen, Lan Li.

**Data curation:** Guowei Chen.

**Formal analysis:** Guowei Chen, Hongguang Pan, Zhenjiang Liang, Lan Li.

**Investigation:** Guowei Chen, Yizhen Luo.

**Project administration:** Guowei Chen.

**Resources:** Guowei Chen, Yizhen Luo.

**Writing – original draft:** Guowei Chen, Yishu Teng, Lan Li.

**Writing – review and editing:** Guowei Chen, Zhenjiang Liang, Lan Li.
